# 1937. COVID-19 Vaccine Distribution in Massachusetts Jails

**DOI:** 10.1093/ofid/ofac492.1564

**Published:** 2022-12-15

**Authors:** Nicole Cassarino, Emma Smyth, Alysse Gail Wurcel

**Affiliations:** Tufts University School of Medicine, Brookline, Massachusetts; Tufts Medical Center, Boston, Massachusetts; Tufts Medical Center, Boston, Massachusetts

## Abstract

**Background:**

Vaccination is one of the main mitigation strategies to protect people from COVID19 mortality. People incarcerated in jails experienced disparate rates of COVID19 infection compared to people in the community, thus operationalization of COVID19 vaccine delivery in jails was prioritized in several states, including Massachusetts (MA). The goal of this project was to track COVID19 vaccine ordering in MA jails and compare numbers and types of COVID19 vaccines ordered by MA jails to those in the MA community, with specific attention to the Centers for Disease Control and Prevention’s (CDC) guidelines for COVID19 vaccines.

**Methods:**

MA jails received COVID19 vaccines free of charge through the MA Department of Public Health. We requested de-identified, facility-level data from the MA DPH including: number of vaccines ordered by each jail, type of vaccine, and date of the vaccine order from December 2020 - January 2022. We obtained COVID19 vaccines distribution data for the MA general population from the CDC.

**Results:**

Vaccine orders were available for 13/14 MA jails. A total of 23,060 vaccines were ordered from the MA DPH between December 2020 - January 2022. January 2021 marked the highest number of vaccine orders by the jails, and all other months were orders < 33% of the January 2021 order. Moderna COVID vaccines were most frequently ordered by the MA jails (88%), followed by Janssen (11%) then Pfizer (1%). In the general population, Pfizer was the most frequently distributed vaccine type (59%), followed by Moderna (37%), then Janssen (4%).

**Figure:**
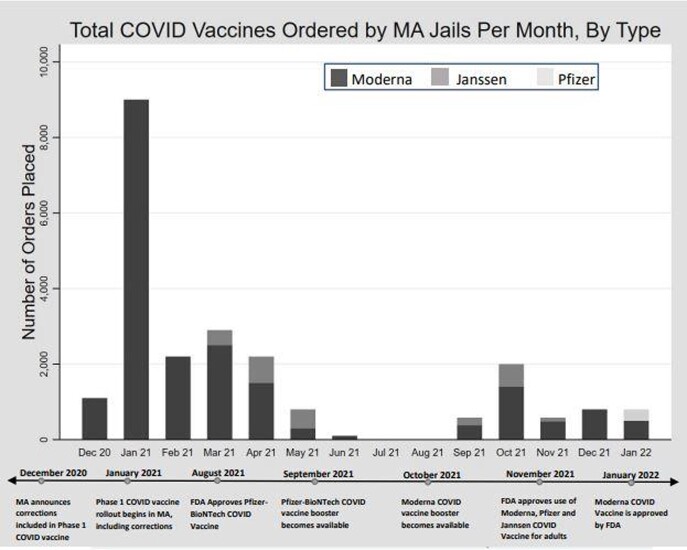

**Figure:**
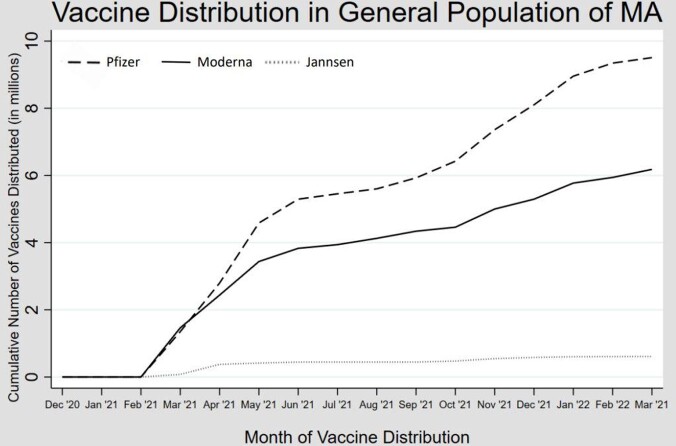

**Conclusion:**

Prioritization of jails in MA led to a strong initial push for vaccine distribution which then waned. Potential reasons for the drop in orders include: the initial vaccine orders in January 2021 may have lasted several months, people entering jail after spring 2021 may have received vaccines in the community, and clinical and administrative fatigue may have limited continued robust vaccine protocols. Tracking COVID-19 vaccine orders by jails is a way to assess equitable vaccine operationalization. As recommendations for vaccination evolve, public health and carceral leaders should collaborate to ensure consistent low-barrier COVID19 vaccine access in jails

**Disclosures:**

**All Authors**: No reported disclosures.

